# Analysis of differentially expressed genes discovers Latroeggtoxin VI-induced changes and *SYNJ1* as a main target in PC12 cells

**DOI:** 10.1186/s12864-023-09634-5

**Published:** 2023-09-04

**Authors:** Dianmei Yu, Haiyan Wang, Zhixiang Lei, Yiwen Zhai, Si Chen, Minglu Sun, Panfeng Yin, Xianchun Wang

**Affiliations:** State Key Laboratory of Developmental Biology of Freshwater Fish, Protein Chemistry Laboratory, College of Life Sciences, Hunan Normal University, Changsha, 410081 Hunan China

**Keywords:** Latroeggtoxin-VI, Differential transcriptome, PC12 cell, Transcript-encoded protein, Dopamine, Synaptojanin 1

## Abstract

**Background:**

Previous preliminary work found that Latroeggtoxin-VI (LETX-VI), a proteinaceous neurotoxin from the eggs of spider *Latrodectus tredecimguttatus*, could promote the synthesis and release of dopamine in PC12 cells. However, the underlying mechanisms have not been fully clear. Here, the effects of LETX-VI on the gene expression profile and dopamine in PC12 cells were analyzed with the differential transcriptome-based strategies.

**Results:**

After treatment of PC12 cells with LETX-VI for 24 h, a total of 356 differentially expressed transcripts were identified. Of them 165 were up-regulated and 191 down-regulated. Relevant GO analysis indicated that LETX-VI modulated the expression of certain genes and thereby affected multiple biological processes in PC12 cells, including protein metabolism, nucleic acid metabolism, substance transport, signaling, neurotransmitter metabolism and release. When western blot analysis was employed to confirm the abundance levels of synaptojanin 1 and synuclein alpha interacting protein, the representatives of highly up- and down-regulated transcript-encoded proteins that are closely related with dopamine respectively, it was found that the level of synaptojanin 1 in the PC12 cells treated with LETX-VI was increased, whereas that of synuclein alpha interacting protein was not obviously altered, suggesting that synaptojanin 1 may be much more involved in the effects of LETX-VI on dopamine. After synaptojanin 1 level was knocked down using siRNA, the levels of both total and released dopamine were significantly decreased, indicating that synaptojanin 1 is a protein positively modulating the synthesis and secretion of dopamine. When the PC12 cells with knocked down synaptojanin 1 were treated by LETX-VI, the adverse effects of synaptojanin 1 knockdown on dopamine were attenuated, confirming that LETX-VI promotes the synthesis and secretion of dopamine at least partially by enhancing the expression of the gene *SYNJ1* encoding synaptojanin 1.

**Conclusions:**

This work demonstrates that LETX-VI exerts multiple regulatory effects on the cellular processes in PC12 cells by altering the gene expression profile. LETX-VI modulates the expression of the genes closely related to the synthesis, transport and release of neurotransmitters especially dopamine in PC12 cells, with the gene *SYNJ1* encoding synaptojanin 1 as a main target.

**Supplementary Information:**

The online version contains supplementary materials available at 10.1186/s12864-023-09634-5.

## Background

*Latrodectus tredecimguttatus* spiders (also named black widow spiders) belong to Theridiidae, Latrodectus in animal taxology [1, 2], and are extensively distributed in much of the world including some provinces of China [2, 3]. The black widow spider is one of the most venomous spider species; its venom secreted by venomous glands contains a diverse series of biologically active components, mainly large proteins and small peptides [3–6]. Interestingly, different from many other venomous animals such as snake, scorpion and some other spider species that have toxins only in their venomous glands, the black widow spiders produce toxic components not only in their venomous glands, but also in their body tissues and even eggs [5, 7]. After the mice received intravenous injections of the extract of decapitated adult spider, the mice became somewhat ill, showing hypoactivity, eyelid closing and some ruffling of the hair [8]. Abdominal injection of the extract of *L. tredecimguttatus* spiderlings into mice and *P. americana* resulted in obvious poisoning symptoms and even death [9]. The extract of the *L. tredecimguttatus* eggs is rich in large proteins and small peptides, showing multiple hydrolase activities and neurotoxicities toward mammals and insects. No known latrotoxins, the typical toxins in the venom of Latrodectus spiders, were found in the egg extract, suggesting that the eggs have the toxicity basis and mechanisms different from those of the venom secreted by venomous glands [10]. Obviously, investigating the toxic components outside the venomous glands not only extends our understanding of spider toxins, but also finds novel lead compounds for the development of tool reagents in scientific research and drugs in the clinic. Up to now, several proteinaceous toxins, named latroeggtoxins, have been characterized from the *L. tredecimguttatus* eggs [11–14]. Latroeggtoxin-VI (LETX-VI) is the sixth proteinaceous toxin found in the *L. tredecimguttatus* eggs.

Previous relevant work demonstrated that LETX-VI could penetrate plasma membrane and enter the nucleus of PC12 cells, a most commonly used dopaminergic neuron model, and promote the synthesis and release of dopamine in PC12 cells [15]. However, the effects of LETX-VI on PC12 cells have not been unraveled completely. The next-generation RNA sequencing technique has characteristics such as high-throughput and low cost, and can identify almost all the mRNAs and their relative abundance in a biological sample. Such a technique is very useful in detecting differential expression of genes and improving function analysis of a biologically active molecule. In the present study, differential transcriptome analysis in combination with other techniques was employed to explore the effects of LETX-VI on the gene expression profile and biological processes in PC12 cells, so as to further understand the biological functions of this neurotoxin.

## Results

### Cytotoxicity of LETX-VI toward PC12 cells

**Fig. 1:**
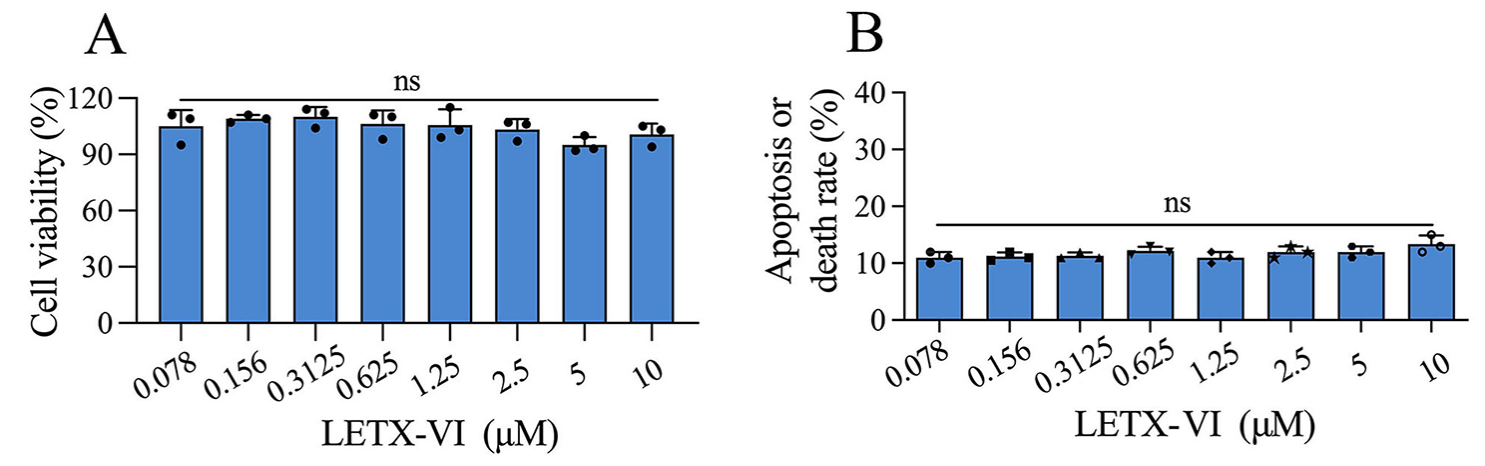
Cytotoxicity of LETX-VI toward PC12 cells. (**A**) Effect of LETX-VI on viability of PC12 cells determined with a CCK-8 kit. (**B**) Apoptosis or death rate of PC12 cells exposed to LETX-VI determined by LDH leakage. ns: not significant

Before we performed the analysis on differential transcriptome of PC12 cells, the cytotoxicity of LETX-VI treatment for 24 h toward PC12 cells was investigated. As shown in Fig. 1A, compared with the control, the viability of PC12 cells was not significantly altered after 24 h treatment with LETX-VI at concentrations up to 10 µM (*P* > 0.05). LDH leakage was also used to detect the cytotoxicity of LETX-VI in this study. If LETX-VI damaged the integrity of plasma membranes of PC12 cells, LDH in the cytoplasm would be released into the culture medium. Figure 1B shows that after exposure to LETX-VI at concentrations up to 10 µM for 24 h, the apoptosis or death rates of PC12 cells, calculated based on the LDH level in the culture medium, was in the range of 11–15%, which were not significantly different from that of the control (*P* > 0.05). These data suggest that the cytotoxicity of LETX-VI toward PC12 cells was limited under the present experimental conditions, and the effects of LETX-VI on PC12 cells were not primarily based on its cytotoxicity but rather on its regulatory actions on various cellular processes. Comprehensively considering the cost and other factors, we used 2.0 µM as the final concentration of LETX-VI when treating PC12 cells for differential transcriptome analysis.

### LETX-VI treatment caused changes in gene expression profile of PC12 cells

After the cDNA libraries prepared from the PC12 cells with or without treatment by LETX-VI were sequenced and quality controlled, on average 5,4150,039 and 58,118,469 clean reads were obtained from the control and treatment groups, respectively. The average read length was about 145 bp. The percentages of bases with the base recognition correct rates of 90%, 99% and 99.9% or more were on average 99.98%, 99.22% and 97.13%, respectively. These results show that the raw sequencing data were strictly quality-controlled before further analysis (Additional file 1).

**Fig. 2:**
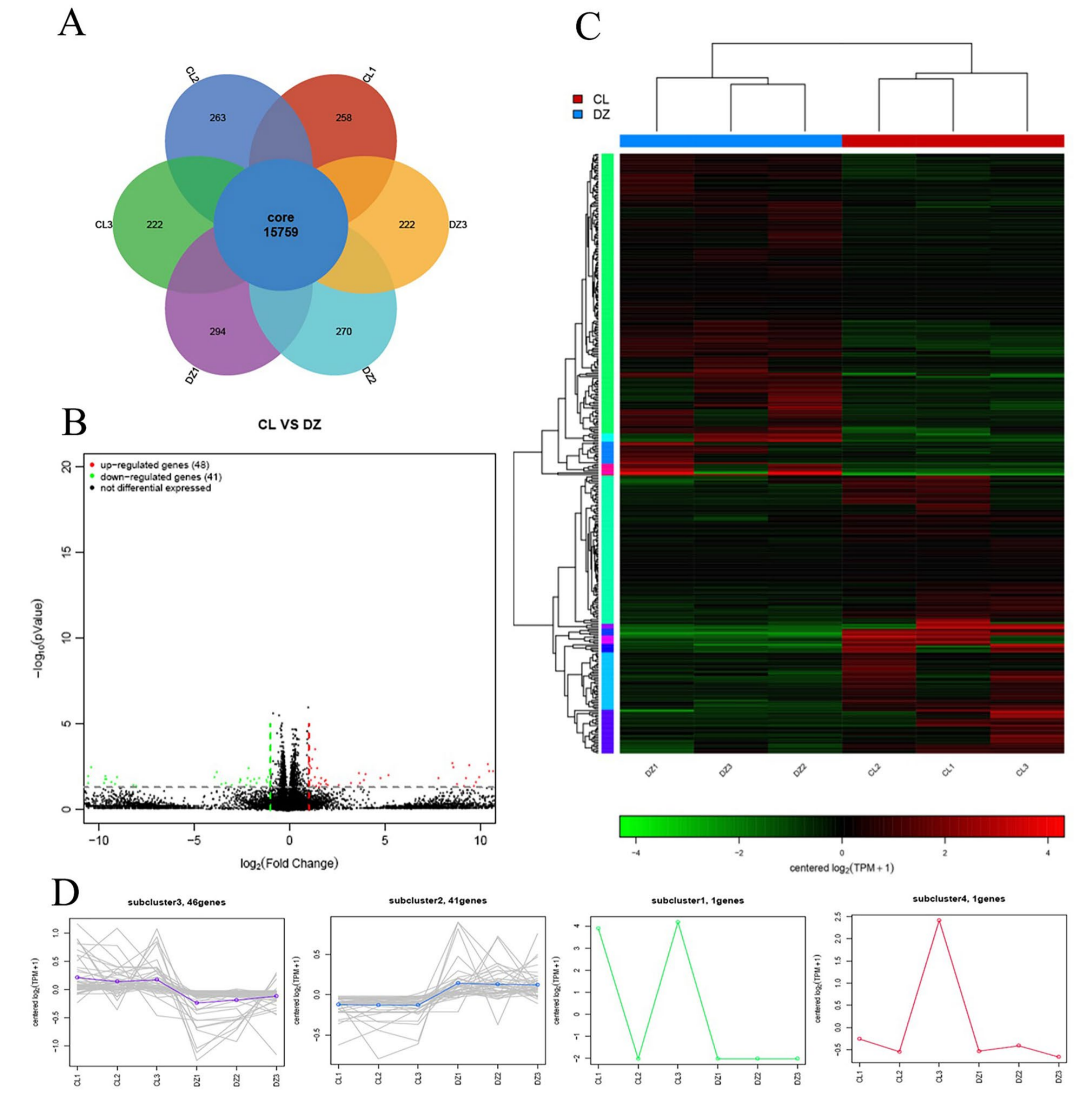
Analysis of the expressed genes identified in PC12 cells. (**A**) Venn diagrams of the genes identified by the 6 CL (LETX-VI treatment) and DZ (control) samples. (**B**) MA plot of differentially expressed genes. TPM, transcripts per kilobase of exon model per million mapped reads. (**C**) Clustering heatmaps of differentially expressed genes. (**D**) Changes in the expression levels of differentially expressed genes in the 6 CL and DZ samples

When mapping the reads to the reference sequences in the used databases (OS = *Rattus norvegicus*), a total of 15,759 expressed genes were identified by the 6 DZ (control) and CL (LETX-VI treatment) samples simultaneously (Fig. 2A). Compared with the DZ group, CL group resulted in the differential expression of 89 genes, of which 48 genes were up-regulated and 41 down-regulated (Fig. 2B). The clustering heatmaps of differentially expressed genes (Fig. 2C) exhibited the changes with different colors in the expression levels of each differentially expressed genes in the 6 samples, showing that there were obvious differences in the gene expression levels between CL and DZ groups. In addition, of the 89 differentially expressed genes, 46 genes were relatively highly expressed in all the 3 CL samples and relatively lowly expressed in all the 3 DZ samples, whereas the expression levels of 41 differentially expressed genes displayed an opposite change law. Only two genes displayed irregular changes in the expression levels of the 6 CL and DZ samples (Fig. 2D), indicating that the expression levels of most differentially expressed genes had good repeatability between the duplicate samples.

**Fig. 3:**
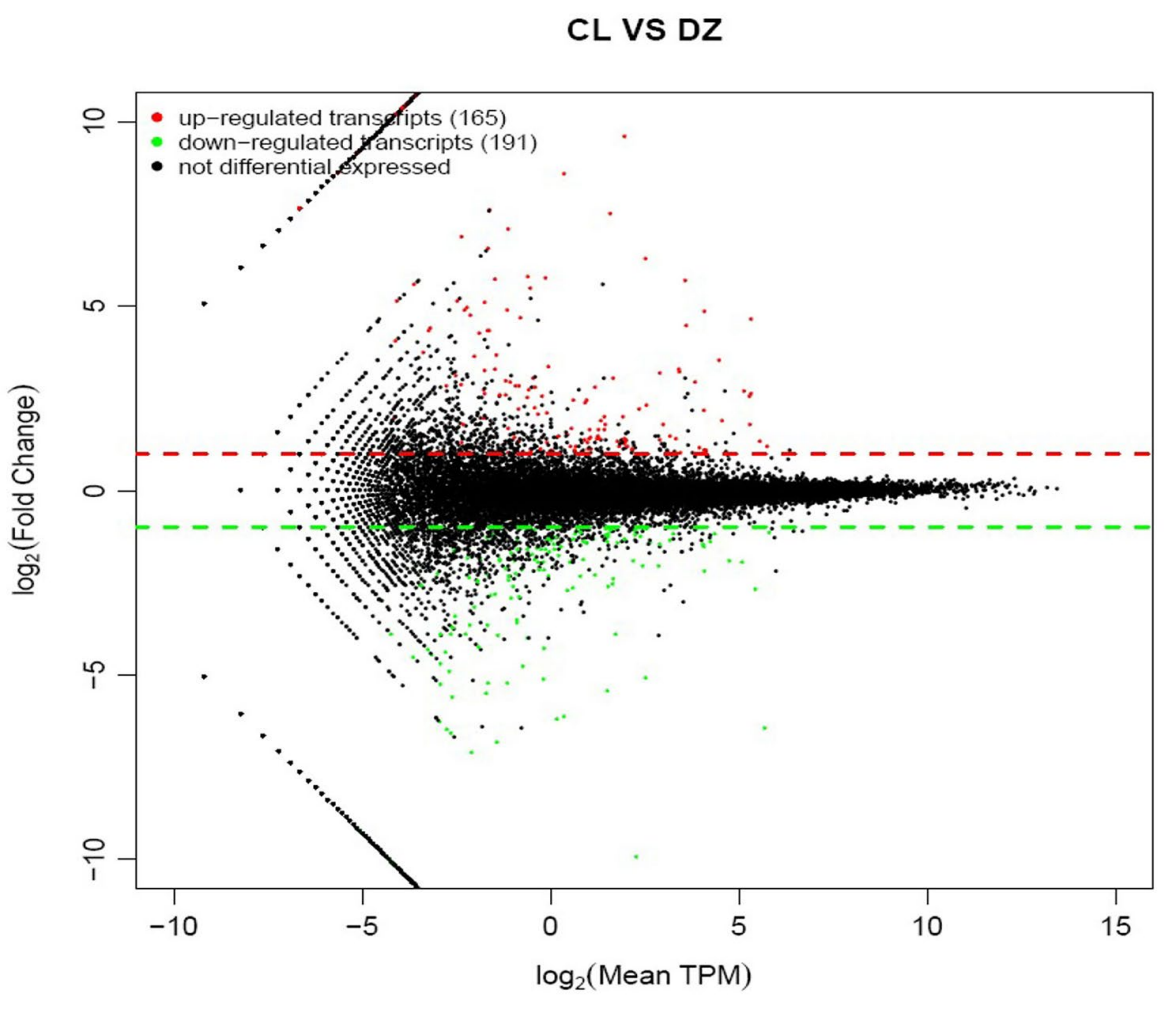
MA plot of differentially expressed transcripts. CL: LETX-VI treatment; DZ: Control

As a result, a total of 40,808 non-redundant transcripts were identified. When the threshold for differential expression was set at 2-fold change (*P* < 0.05), compared with the control, LETX-VI treatment led to 356 transcripts being significantly differentially expressed, among which 165 were up-regulated and 191 down-regulated (Additional files 2 and 3). From the Fig. 3, it can be seen that the log2(Fold change) values of differentially expressed transcripts were primarily distributed from about − 5 to 5, and the log2 (Mean TPM) values were mainly distributed from about − 5 to 10, demonstrating that LETX-VI led to obvious changes in the expression levels of certain transcripts in PC12 cells.

### Interaction network analysis of differentially expressed transcript-encoded proteins

**Fig. 4:**
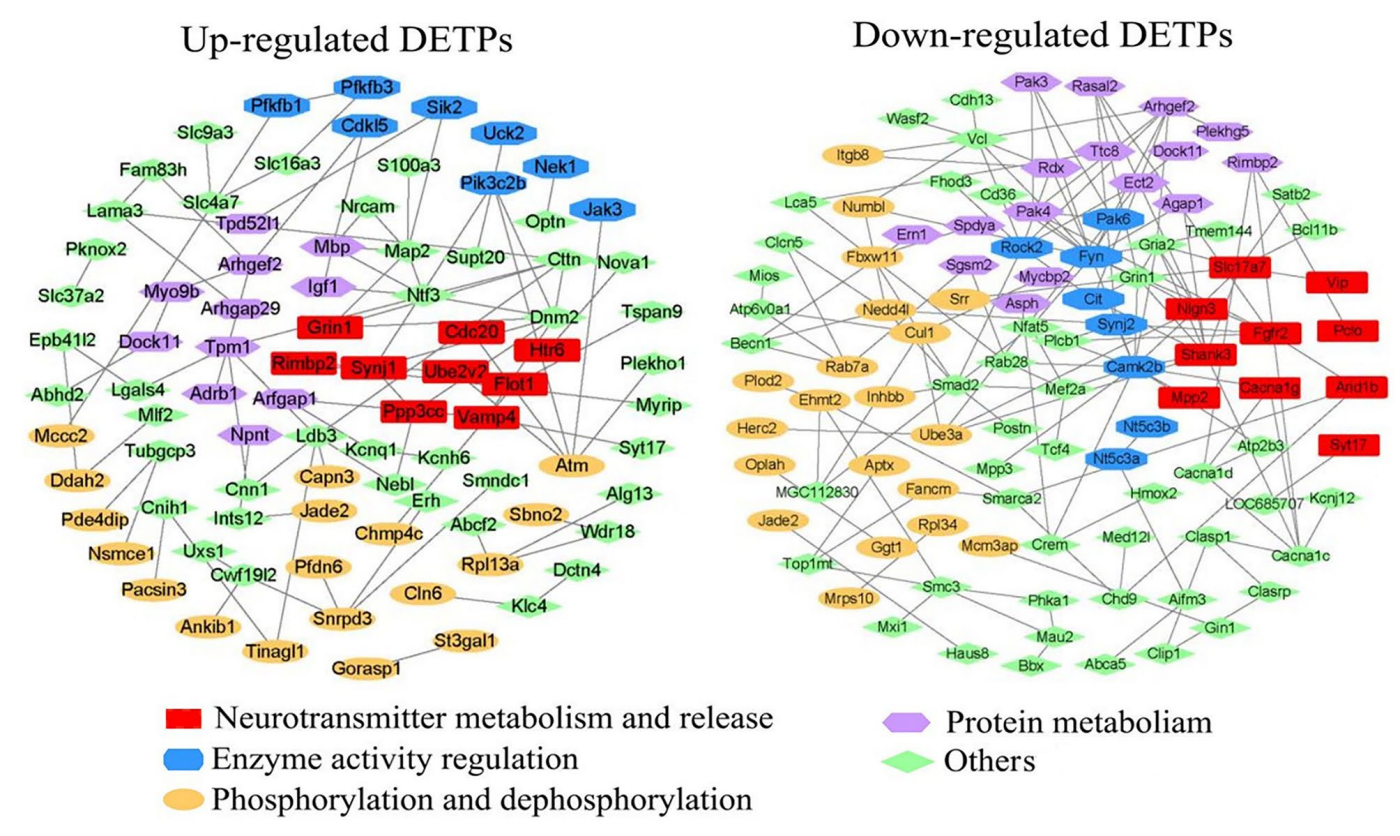
Interaction network analysis of DETPs.

After LETX-VI treatment, the transcript levels of certain genes were significantly altered, indicating that LETX-VI could influence the metabolic processes in PC12 cells through changing gene expression and protein abundance, although the level of a protein is not always parallel to that of the respective transcript [16]. In order to further probe into the effects of LETX-VI on PC12 cells, we made a systematic analysis of the differentially expressed transcript-encoded proteins (DETPs). When we performed the interaction network analysis of DETPs using their encoding genes through the STRING and Cytoscape softwares (https://cn.string-db.org/ and https://cytoscape.org/), with the genes without interactions being automatically removed, it was found that there were extensive interactions between most of the DETPs. The DETPs are involved in multiple signaling pathways and cellular processes, such as neurotransmitter metabolism and release, enzyme activity regulation, phosphorylation and dephosphorylation, protein metabolism, etc. (Fig. 4). The functionally correlative proteins form clusters that are labeled with different colors in the figure. The proteins in a cluster tend to interact with each other and many proteins in different clusters may also have interactions.

### GO analysis of DETPs

#### Subcellular distribution of DETPs

**Fig. 5:**
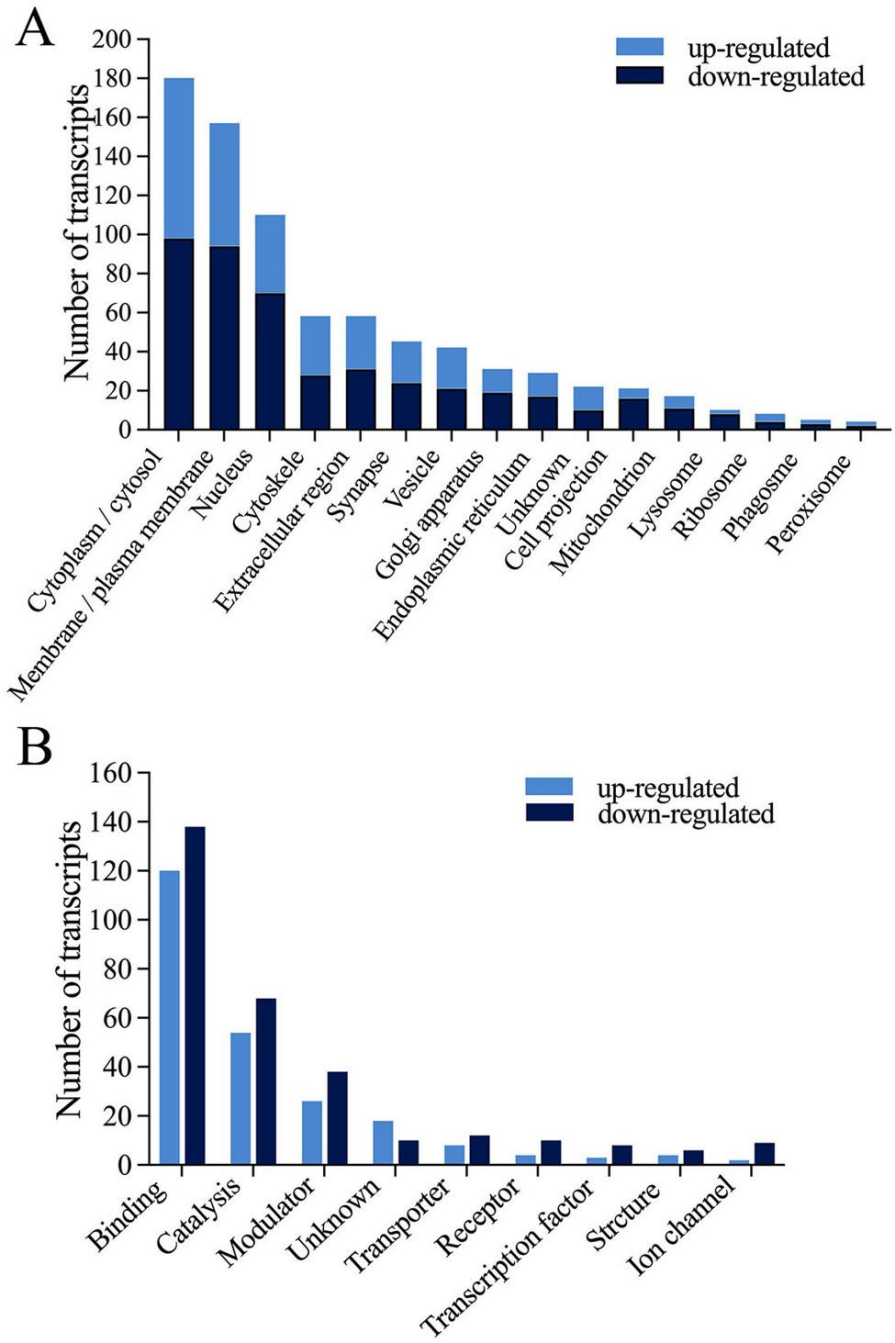
Subcellular distribution (**A**) and molecular function (**B**) of DETPs.

When we analyzed the cellular components for the DETPs, about 1540 GO cellular component terms were retrieved from the rat-related databases. For the sake of brevity, we classified the cellular components of these proteins into 15 categories according to the relevance of the terms: cytoplasm/cytosol, membrane/plasma membrane, nucleus, cytoskeleton, extracellular region, synapse, vesicle, Golgi apparatus, endoplasm reticulum, cell projection, mitochondrion, lysosome, ribosome, phagosome, peroxisome. In addition, 24 DETPs (accounting for 6.74% of 356) had no GO cellular component terms in the available databases and were classified into the category of unknown (Fig. 5A; Additional files 2 and 3). A protein usually has one or more subcellular locations and thus was grouped into one or more categories. Statistics showed that cytoplasm/cytosol was the main subcellular location for DETPs, with 180 DETPs (accounting for 50.56%) being distributed in this subcellular compartment, followed by membrane/plasma membrane (157, 44.10%) and nucleus (109, 30.62%), demonstrating that the proteins in these subcellular compartments were relatively more susceptible to the effect of LETX-VI.

#### Molecular function of DETPs

For understanding the molecular functions of DETPs, the universal GO molecular function annotations were searched in the rat-related databases, and as a result in total about 1220 terms were retrieved. We classified these terms into 8 categories according to their functional similarity: binding, catalysis, modulator, transporter, receptor, transcription factor, structure, ion channel. Twenty-eight DETPs (accounting for 7.87%) without GO molecular function annotations were classified into the category of unknown (Fig. 5B; Additional files 2 and 3). DETPs with the molecular function of binding constituted the largest category, with more than 70% DETPs (255, 71.63%) being classified into this category. These proteins could affect a series of cellular processes by binding to relevant cellular components such as ubiquitin conjugating enzyme, calmodulin, syntaxin, neurotransmitter, acetylcholine receptor, transcription factor, GTPase activator, Ca^2+^, ATP and NAD^+^. The second largest category (120, 33.71%) was composed of enzymes with catalytic function. These enzymes catalyzed multiple biochemical reactions involved in substance and energy metabolisms as well as their regulation. These results demonstrate that changing the expression levels of enzymes was one of the major action mechanisms for LETX-VI to affect the PC12 cells.

Sixty-one DETPs (accounting for 17.13%) were classified into the category of modulator, as they had activities of transcription activator, transcription repressor, splicing regulator, translation initiation factor, enzyme inhibitor, hormone, and so on. These DETPs modulate the activities of regulatory factors and thus participate in the regulation of cellular metabolism. Besides, the DETPs with function of transporter, receptor, transcription factor, ion channel and structure molecule accounted for 22%, 18%, 15%, 15% and 13%, respectively. Although the proportions of the DETPs in these several categories were relatively lower, they were speculated to also play important roles in the effects of LETX-VI on PC12 cells.

#### Biological processes involved in DETPs

**Fig. 6:**
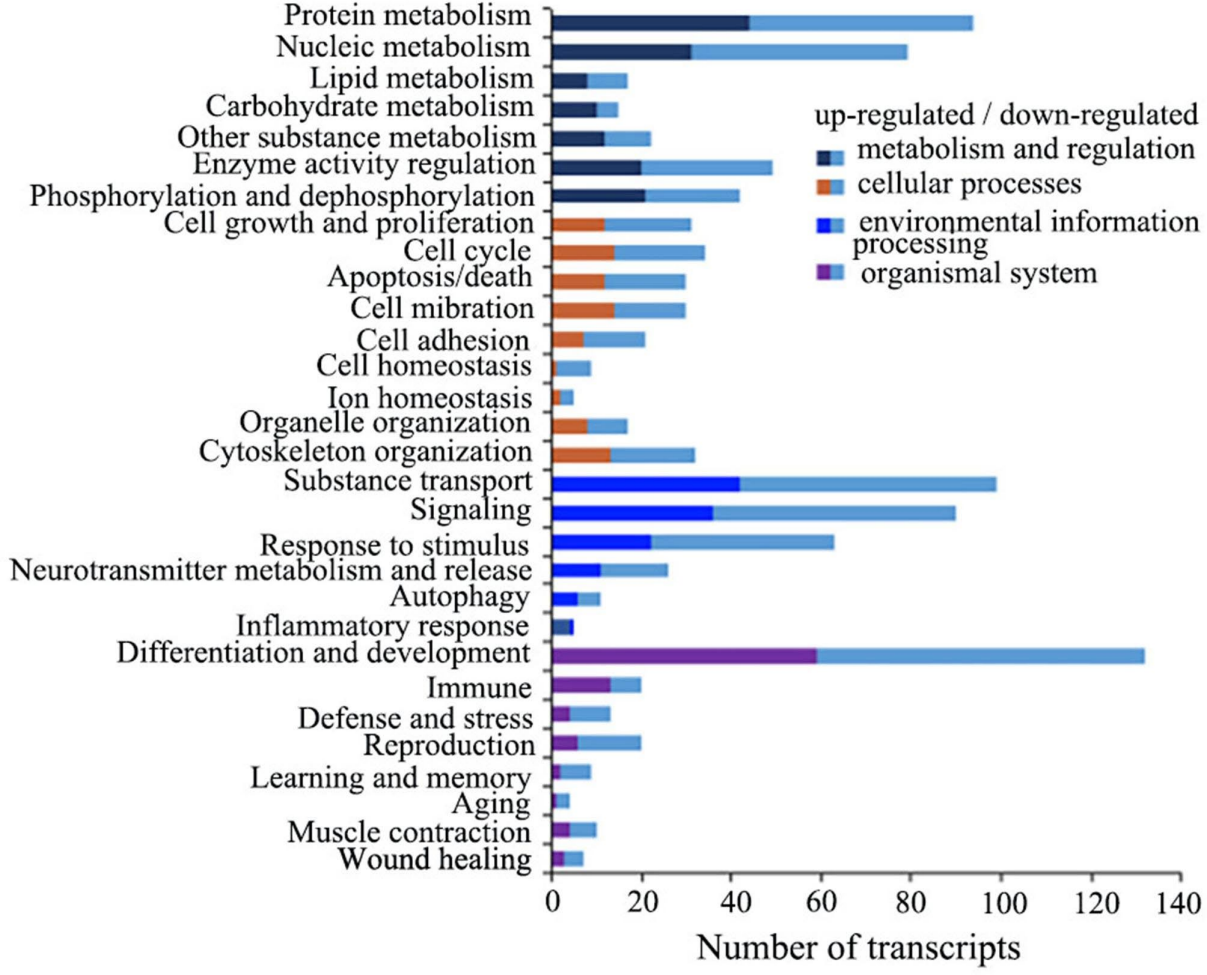
Biological processes involved in DETPs.

A total of about 2400 biological process GO terms were retrieved from the relevant databases. For the convenience of analysis, the biological processes that the DETPs were involved in were categorized into 30 groups according to the correlation of the terms: protein metabolism, nucleic acid metabolism, enzyme activity regulation, cell growth and proliferation, cell cycle, apoptosis, substance transport, signaling, neurotransmitter metabolism and release, differentiation and development, etc., which were divided into 4 major groups: metabolism and regulation, cellular processes, environmental information processing and organismal systems. In addition, 27 DETPs (accounting for 7.58%) had no GO biological process terms in the rat-related databases and were categorized into the group of unknown (Fig. 6; Additional files 2 and 3).

**Fig. 7:**
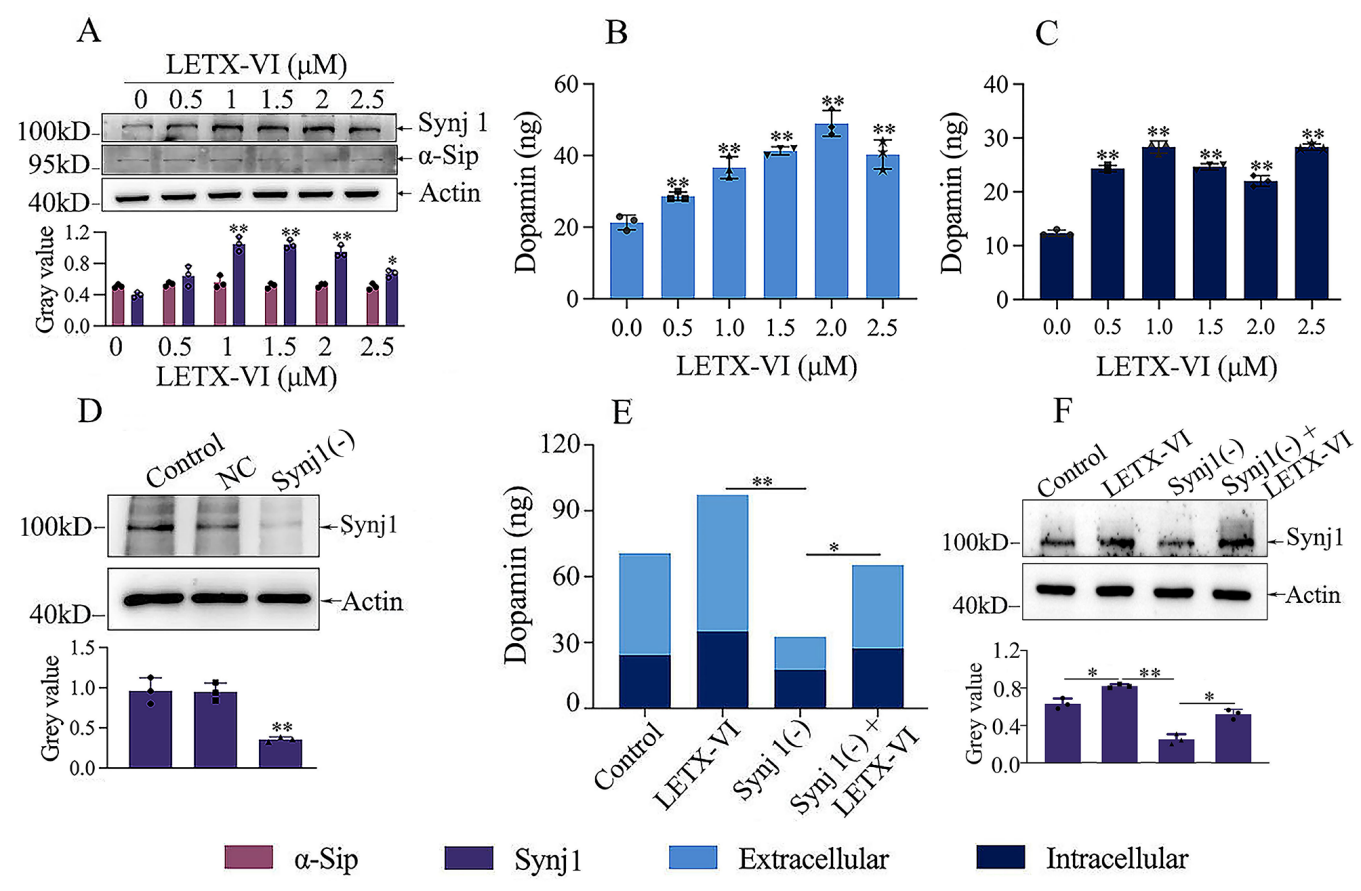
Mediation of synaptojanin 1 on the LETX-VI-caused increase in dopamine level. (**A**) Effects of LETX-VI at different concentrations on the levels of synaptojanin 1 (Synj1) and synuclein alpha interacting protein (α-Sip).*P < 0.05 and ** *P* < 0.01 vs. control. The uncropped full-length blot is included in the Additional file 4. (**B**) and (**C**) Effects of LETX-VI at different concentrations on the levels of extracellular and intracellular dopamine, respectively. ** *P* < 0.01 vs. control. (**D**) Efficiency of synaptojanin 1 (Synj1) knockdown detected by western blot analysis. NC: negative control. Synj1 (-): PC12 cells with synaptojanin 1 knockdown by siRNA. **P < 0.01 vs. control and NC. The uncropped full-length blot is included in the Additional file 4. (**E**) Effects of LETX-VI and synaptojanin 1 knockdown on dopamine level of PC12 cells. LETX-VI: treatment of wild- type PC12 cells with 2.0 µM LETX-VI. Synj1 (-): PC12 cells with knocked down synaptojanin 1. Synj1 (-) + LETX-VI: treatment of PC12 cells with knocked down synaptojanin 1 by 2.0 µM LETX-VI. * *P* < 0.05, ** *P* < 0.01. (**F**) Effects of LETX-VI and synaptojanin 1 knockdown on the synaptojanin 1 protein level of PC12 cells. LETX-VI: treatment of wild-type PC12 cells with 2.0 µM LETX-VI. Synj1 (-): PC12 cells with knocked down synaptojanin 1. Synj1 (-) + LETX-VI: treatment of PC12 cells with knocked down synaptojanin 1 by 2.0 µM LETX-VI. The uncropped full-length blot is included in the Additional file 4. * *P* < 0.05, ** *P* < 0.01. The data are presented as the mean ± SD.

As shown in Fig. 7, 94 DETPs in the category of protein metabolism accounted for 26.40%. They participate in various aspects of protein and amino acid metabolisms including their regulation, such as translation, post-translational protein modification, protein folding, proteolysis, protein ubiquitination, synthesis and degradation of amino acids, indicating that the protein metabolism in the PC12 cells was greatly influenced by the LETX-VI in terms of the number and impact range of differentially expressed transcript-encoded proteins. Likewise, nucleic acid metabolism was also obviously affected by LETX-VI, as the expression level of the transcripts for 79 DETPs (22.19%) involved in nucleic acid metabolism was significantly up-regulated or down-regulated. Comparatively, lipid and carbohydrate metabolisms were less influenced by LETX-VI. Besides, in the major group “Metabolism and regulation” there were 49 (13.76%) and 42 (11.80%) DETPs that could regulate enzyme activity and affect phosphorylation and dephosphorylation reactions, demonstrating that modifying the regulatory processes was also one of the main action mechanisms for LETX-VI to influence the substance and energy metabolisms in PC12 cells.

The major group “Cellular processes” mainly contained the DETPs that influence the viability and physiological state. Although the DETPs influence multiple aspects of PC12 cell states, the involved DETPs were relatively fewer, all less than 10%. For example, the DETPs related with cell growth and proliferation, cell cycle, cell apoptosis/death only accounted for 8.71%, 9.55% and 8.43%, respectively. It was speculated that the effects of LETX-VI on the viability and physiological state of PC12 cells might be relatively limited under the present experimental conditions based on the number of DETPs, although the effects were not completely depended on the number of the DETPs. Furthermore, the cell viability assay supported this speculation (Fig. 1).

In consideration of the fact that LETX-VI was shown to be a neurotoxin and could promote the synthesis and release of dopamine of PC12 cells in the previous work [15], we specially analyzed the DETPs involved in neurotransmitter metabolism and release. Totals of 26 DETPs were categorized in the classification “neurotransmitters metabolism and release”. Among their transcripts, 11 were up-regulated and 15 down-regulated (Fig. 6; Additional files 2 and 3). The representatives of them are listed in Table 1. The table shows that the DETPs, such as lymphocyte antigen 6 family member E, synaptojanin 1, vesicular glutamate transporter 1, synuclein alpha interacting protein (also named synphilin-1), etc., were highly differentially expressed and closely involved in the metabolism, transport and secretion of neurotransmitters as well as synaptic vesicle endocytosis [17, 18].

**Table 1:** Information on representative DETPs involved in neurotransmitter metabolism and release

Transcript ID	log2(Fold Change)	DETP name	Biological process
**Up-regulated:**			
ENSRNOT00000077022	7.10	lymphocyte antigen 6 family member E	norepinephrine metabolic process
ENSRNOT00000002812	5.13	synaptojanin 1	neurotransmitter transport, synaptic vesicle transport, synaptic vesicle priming, synaptic vesicle endocytosis
ENSRNOT00000012993	4.33	serine/threonine-protein phosphatase	positive regulation of synaptic vesicle endocytosis
ENSRNOT00000070865	2.55	5-hydroxytryptamine receptor 6	positive regulation of dopamine secretion, negative regulation of gamma-aminobutyric acid and acetylcholine secretion
ENSRNOT00000082523	1.12	flotillin 1	positive regulation of synaptic transmission (dopaminergic), regulation of neurotransmitter uptake
ENSRNOT00000076922	1.10	vesicle-associated membrane protein 4	regulation of synaptic vesicle endocytosis, synaptic vesicle to endosome fusion
**Down-regulated**:			
ENSRNOT00000025119	-11.40	synuclein, alpha interacting protein	regulation of neurotransmitter secretion, dopamine metabolic process
ENSRNOT00000064184	-10.87	vesicular glutamate transporter 1	synaptic transmission (glutamatergic), neurotransmitter loading into synaptic vesicle, regulation of synaptic vesicle endocytosis
ENSRNOT00000077308	-10.55	histamine receptor H3	regulation of neurotransmitter levels
ENSRNOT00000022331	-10.15	fibroblast growth factor receptor 2	synaptic vesicle transport
ENSRNOT00000007608	-9.23	piccolo (presynaptic cytomatrix protein)	synaptic vesicle exocytosis, synaptic vesicle clustering, synapse assembly
ENSRNOT00000092672	-6.21	SH3 and multiple ankyrin repeat domains 3	positive regulation of synaptic transmission (glutamatergic)
ENSRNOT00000092246	-2.90	synaptotagmin 17	regulation of dopamine secretion
ENSRNOT00000076412	-2.05	lymphocyte antigen 6 complex, locus E	norepinephrine metabolic process

### Validation of representative DETPs closely related with dopamine

For further investigating the effects of LETX-VI on dopamine of PC12 cells, we used western blot analysis to determine the abundance levels of representative DETPs. In view of the fact that protein lymphocyte antigen 6 family member E is mainly involved in the metabolic process of norepinephrine, a minor catecholamine in PC12 cells, we took synaptojanin 1 and synuclein alpha interacting protein as the representatives of up- and down-regulated DETPs, respectively. These two DETPs were closely related to dopamine and had high up-regulated or down-regulated expression folds (Table 1; Additional files 2 and 3).

The results shown in Fig. 7A reveal that, after treatment of PC12 cells with different concentrations of LETX-VI for 24 h, the content of synaptojanin 1 in the PC12 cells was increased, whereas that of synuclein alpha interacting protein was relatively low and was not obviously altered. These results demonstrate that LETX-VI is able to up-regulate the expression of synaptojanin 1 at both transcription and translation levels, and the expression of synuclein alpha interacting protein was not affected at protein level although the transcription of its encoding gene was remarkably down-regulated by LETX-VI. Synuclein alpha interacting protein was thus speculated not to play a significant role in the effects of LETX-VI on dopamine at protein level.

When we detected the LETX-VI-caused changes in extracellular and intracellular dopamine levels, as shown in Fig. 7B and C, treatment of PC12 cells with different concentrations of LETX-VI was found to result in an increase in both extracellular and intracellular dopamine levels, indicating that LETX-VI promoted the synthesis and release of dopamine in PC12 cells. In view of the results that treatment of PC12 cells with LETX-VI led to a significant increase in the expression of *SYNJ1*, the gene encoding synaptojanin 1 (Fig. 7A**)**, and enhanced the synthesis and release of dopamine (Fig. 7B and C), synaptojanin 1 was speculated to mediate the effects of LETX-VI on dopamine. To validate this speculation and further probe into the relationship between the up-regulated synaptojanin 1 and dopamine, we knocked down synaptojanin 1 expression using siRNA, followed by knockdown efficiency detection and dopamine determination. The western blot analysis results shown in the Fig. 7D indicate that synaptojanin 1 was efficiently knocked down. Figure 7E shows that, after synaptojanin 1 was knocked down, the amounts of both extracellular and intracellular dopamine were significantly decreased, and however the decrease amplitude was remarkably reduced when 2.0 µM LETX-VI was applied (*P* < 0.01). For further understanding the effect of LETX-VI on synaptojanin 1, we detected the effects of LETX-VI and synaptojanin 1 knockdown on the synaptojanin 1 protein level of PC12 cells with western blot analysis. The results (Fig. 7F) showed that, after RNA inteference, synaptojanin 1 protein expression in the PC12 cells was significantly decreased compared with the control (*P* < 0.05); however, when the PC12 cells with knocked down synaptojanin 1 was treated with 2.0 µM LETX-VI, the synaptojanin 1 protein level was significantly up-regulated (*P* < 0.05), although it did not reach the levels of the control and LETX-VI groups. These results demonstrate that *SYNJ1* is one of the main target genes for LETX-VI and synaptojanin 1 at least partially mediates the promoting effects of LETX-VI on the synthesis and release of dopamine in PC12 cells.

## Discussion

The incomparable advantages of RNA sequencing, such as high throughput, low cost and high sensitivity, make it a convenient means to preliminarily probe into the mechanism of action of a biologically active molecule. When we investigated the effects of the neurotoxin LETX-VI on PC12 cells, we performed a differential transcriptome analysis of the PC12 cells after treatment by LETX-VI. Cytotoxicity analysis showed that the effects of LETX-VI on PC12 cells were not based on its cytotoxicity but rather on its regulatory actions under the present experimental conditions, which was reflected in the limited cytotoxicity or cell death rate (Fig. 1). However, obvious changes in the expression levels of certain genes in PC12 cells were observed after exposure to LETX-VI. In order to understand on the overal level the effects of LETX-VI on the cellular processes in PC12 cells, we made a systematic interaction network analysis of the DETPs (Fig. 4). The results showed that there were extensive interactions between most of the DETPs. In addition, most DETPs exerted their biological function(s) synergically by interacting with other proteins This suggests that LETX-VI affects cellular processes extensively by changing gene expression and by the interactions among the DETPs. The proteins in the same cluster interact with each other to cooperatively accomplish a particular metabolic or regulatory process. In a cell, different cellular processes are usually linked to each other via proteins/enzymes or intermediates. Therefore, many DETPs in different clusters may also have interactions. These results indicate that LETX-VI has influence on multiple cellular processes in the PC12 cells, such as neurotransmitter metabolism and release, which includes dopamine synthesis and release.

GO (Gene Ontology) describes gene products with three independent categories: cellular component, molecular function and biological process. Go analysis allows us to rapidly understand the charaterestics of DETPs and see the overall picture of the effects of LETX-VI on the PC12 cells. GO analysis indicated that the DETPs are distributed in the distinct cellular compartments, have multiple molecular functions and involve a series of biological processes particularly protein metabolism, nucleic acid metabolism, substance transport, signaling, differentiation and development in terms of the number of DETPs. These observations demonstrate that LETX-VI exerts multiple regulatory effects on the cellular processes in PC12 cells by regulating the expression of relevant genes. Particularly, from the GO analysis, we could find the DETPs that are involved in the dopamine synthesis and release, such as those that play important roles in the synthesis, transport and secretion, etc. (Table 1). The proteins/enzymes that are reponsible for the synthesis and transport of dopamine are mostly localized in the cytosol, vesiclar membrane and plasma membrane. Therefore, GO analysis, including subcellular distribution and biological process, helped us to understand the dopamine-related DETPs even better.

**Fig. 8:**
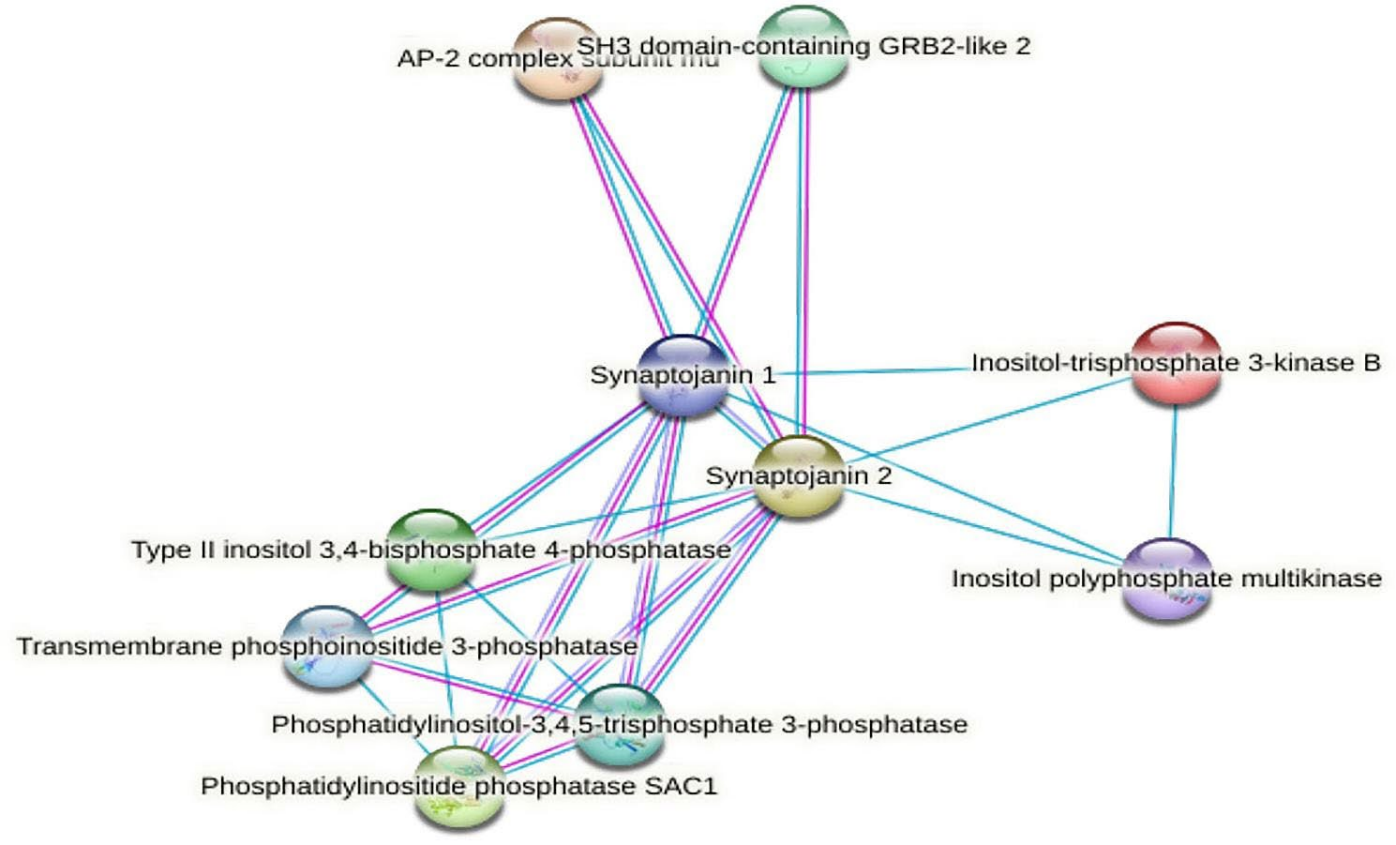
Syanptojanin 1 interaction proteins

In our present study, in view of the close relationship between the functions of LETX-VI and dopamine, we paid a special attention to the DETPs involved in the metabolism, transport and release of the dopamine in PC12 cells. As shown in Table 1, of the significantly up-regulated transcripts that are related with dopamine, the transcript for synaptojanin 1 has an up-regulation fold second only to that for lymphocyte antigen 6 family member E that is mainly involved in the metabolic process of norepinephrine, a minor component in PC12 cells [19, 20]. Synaptojanin 1 is the major synaptic phosphoinositide phosphatase, playing an important role in synaptic vesicle endocytosis and re-availability that ensure maintenance of neurotransmission as well as structural integrity of synapses [20–22]. Phosphatidylinositol-4,5-bisphosphate (PI(4,5) P2) dephosphorylation is essential for the proper recycling of synaptic vesicles at neuronal synapses, whereas synaptojanin 1 is the main PI (4,5) P2 5-phosphatase in the brain [23]. Besides, synaptojanin 1 interacts with multiple phosphoinositol metabolism-related proteins such as SH3 domain-containing GRB2-like 2, AP-2 complex subunit mu, inositol-trisphosphate 3-kinase B to promote synaptic vesicle uncoating, vesicle budding from membrane, signal transduction, neuron protection, etc. (Fig. 8). Genetic deletion of synaptojanin 1 in neurons leads to synaptic transmission defects [24, 25]. Cleavage of synaptojanin 1 by asparagine endopeptidase leads to presynaptic dysfunction and dopaminergic neuronal vulnerability [26]. Our present study showed that LETX-VI treatment of PC12 cells led to increased levels of synaptojanin 1 and dopamine, which suggests that syanptojanin 1 was related with the effects of LETX-VI on dopamine. This suggestion was further confirmed by knockdown of syanptojanin 1 (Fig. 7). Combining our experimental results and relevant literature reports, syanptojanin 1 at least partially mediates the promoting effects of LETX-VI on dopamine by facilitating vesicle endocytosis and re-availability, and protecting dopaminergic neurons synthesizing dopamine. Of course, in addition to synaptojanin 1, some other up-regulated DETPs are also speculated to participate in the effects of LETX-VI on dopamine. For example, serine/threonine-protein phosphatase and vesicle-associated membrane protein 4 [27, 28]; 5-hydroxytryptamine receptor 6 positively regulates dopamine secretion and synaptic transmission [29].

Of the significantly down-regulated transcript-encoded proteins, synuclein alpha interacting protein also attracted our attention due to its largest down-regulation fold and close relationship with dopamine (Table 1). However, western blot analysis indicated that, although the transcript for synuclein alpha interacting protein was greatly down-regulated by LETX-VI, the level of synuclein alpha interacting protein was not obviously altered after LETX-VI treatment. This suggests that, unlike *SYNJ1* gene, the gene for synuclein alpha interacting protein was not heavily involved in the action of LETX-VI on dopamine at protein level. Nevertheless, other DETPs with significantly down-regulated transcripts are speculated to be also involved in the effects of LETX-VI on dopamine to different degrees, although they were not further confirmed one by one in our present study.

## Conclusion

In the present study, the proteinaceous neurotoxin LETX-VI has been shown to possess limited cytotoxicity torward PC12 cells, a commonly used neuronal cell model, and however to alter the transcription of certain genes in PC12 cells, thereby affecting multiple biological processes in the cells, particularly protein metabolism, nucleic acid metabolism, substance transport, signaling, neurotransmitter metabolism and release, etc. LETX-VI affected dopamine in PC12 cells by regulating the expression of the genes closely related to the synthesis, transport and release of dopamine, particularly the gene *SYNJ1* encoding synaptojanin 1. These observations on LETX-VI-caused DETPs and their effects on neurotransmitters particularly dopamine not only extended our understanding of the biological functions of LETX-VI, but also provided guidance for its further researches, including its potential applications in the development of the pharmacological research tools and the drugs to treat dopamine deficiency-related diseases such as depression and Parkinson’s disease.

## Materials and methods

### Effect of LETX-VI on the viability of PC12 cells

Preparation of recombinant LETX-VI was performed according to the previous method [15]. For evaluating the effect LETX-VI on the viability of PC12 cells purchased from the Cell Bank of Type Culture Collection of the Chinese Academy of Sciences, Shanghai, China, a Cell counting Kit-8 (Beyotime Biotechnology Co., Ltd., Shanghai, China) was employed. PC12 cells were seeded in Dulbecco’s modified Eagle’s medium (DMEM) containing 10% fetal bovine serum (FBS) in a 96-well plate and incubated at 37 ^o^C in an incubator with a humidified atmosphere of 5% CO_2_ and 95% air. When the cells reached 80–90% confluence, the culture medium was removed by aspiration and LETX-VI diluted with serum-free DMEM medium was added to the wells at different concentrations (0, 0.078, 0.156, 0.3125, 0.625, 1.25, 2.5, 5, 10 µM), each of which was added to 3 wells. After a 24-h continuous incubation, 10 µL CCK-8 reagent was added into each well and the plate was incubated in dark for about 2 h at 37 ^o^C till the brown color was produced, followed by absorbance measurement at 450 nm on a Multimode Plate Reader (EL×800, BioTek Instruments, Inc.).

To further understand the possible cytotoxicity of LETX-VI toward PC12 cells, the activity of lactate dehydrogenase (LDH) released from PC12 cells into culture medium after LETX-VI treatment was determined using a LDH Cytotoxicity Assay Kit (Beyotime Biotechnology Co., Ltd., Shanghai, China). In brief, PC12 cells were seeded in a 96-well plate and incubated till the the cells were grown to reach 80–90% confluence. The wells were divided into 4 groups: blank, control, maximum LDH activity and LETX-VI treatment, each group consisting of 3 wells. After removal of the culture medium, the cells in LETX-VI treatment group were treated with LETX-VI prepared with FBS-free DMEM to different final concentrations (0.078, 0.156, 0.3125, 0.625, 1.25, 2.5, 5, 10 µM) for 24 h. The culture medium was collected to determine the activity of released LDH according to the manufacturer’s instructions. The percent of cytotoxicity or death rate was determined using the following equation: Cytotoxicity or death rate (%) = (LETX-VI-treated sample OD_490_ − control sample OD_490_) / (maximum LDH activity sample OD_490_ − control sample OD_490_) ×100%.

### PC12 cell culturre and LETX-VI treatment for transcriptome analysis

PC12 cells were subcultured in six 10-cm culture dishes in DMEM supplemented with 10% FBS and 1% penicillin/streptomycin. The cell culture was performed in an incubator at 37 ^o^C in an atmosphere of 5% CO_2_ in air and 100% relative humidity. Three of the culture dishes were used as control groups and the rest as test groups. When the cells were grown to 80–90% confluence, LETX-VI was separately added into the three culture dishes of the test groups (final concentration 2.0 µM) to treat the cells for 24 h. All the control and treated cells were collected and total RNA was extracted with a Total RNA Extractor (Trizol) Kit (Sangon Biotech Co., Ltd. Shanghai, China) according to the manufacture’s protocol and used for transcriptome analysis.

### Transcriptome sequencing and bioinformatics

After the total PC12 cell RNA was treated with DNase I and went through rigorous quality control, poly (A) mRNA was purified from the total RNA using the VAHTS™ mRNA-seq V2 Library Prep Kit for Illumina® (Sangon Biotech Co., Ltd. Shanghai, China). Following purification, the mRNA was randomly broken into small fragments using divalent cations under elevated temperature in fragmentation buffer. These RNA fragments were used as the template to synthesize the first strand cDNA by reverse transcription with random primers. The second strand cDNA was synthesized by DNA polymerase I after RNase H treatment. The cDNAs were end repaired, A-tailed and ligated to sequencing adapters. These cDNA products were purified and the products of 200–500 bp were selected to be amplified by PCR to create the final cDNA library for sequencing.

The cDNA library was sequenced on the Illumina Hiseq^™^ 2000. Both ends of each DNA fragment were determined. Quality of the generated reads was checked using a FastQC software. The raw reads were cleaned by removing the sequences with unknown base (N), adaptor sequences, low quality bases (Q value < 20) and other types of unwanted sequence such as the sequence of less than 35 nucleotides with software Trimmomatic [30]. DESeq2 R package (version 1.12.4) was used to analyze the differentially expressed genes [31]. Enrichment analysis was performed using the topGO R pakage (version 2.24.0). The databases NCBI NR, NCBI NT, GO, KEGG, NCBI COG/KOG, Uniprot, Ensembl, Biomart, PFAM, CDD and STRING were involved in the bioinformatic analyses.

### Knockdown of synaptojanin 1 by RNA interference

In order to knock down synaptojanin 1 level with RNA interference, the interfering RNA sequence 5′ -GGCGAAGUUAUACUUAUAATT-3′ (sense chain) and 5′ -UUAUAAGUAUAACUUCGCCTT-3′ (antisense chain) were chemically synthesized. At the same time, the scrambled sequences were also synthesized as the negative control. The siRNA powder was dissolved in diethyl pyrocarbonate (DPEC)-treated water and prepared into a 20 µM solution. For transfection into PC12 cells, the cells were passaged in 6-well plates to be cultured in DMEM medium containing 10% FBS. Once 75–85% of cell confluence was reached, original culture medium was replaced with the DMEM medium without serum. 4 µl of 20 µM siRNA and 8 µl lipofectamin 2000 were mixed with 200 µl serum-free DMEM medium, followed by thorough gently mixing and incubation at room temperature for 30 min in order to form siRNA/Lipofectamin 2000 complexes. The formed complexes were added into the cell culture medium in the 6-well plates and cultured in a 5% CO_2_ incubator at 37 ℃ for 10 h, and then the culture medium was replaced with DMEM medium containing 10% FBS, followed by culturing for another 38 h and western blot analysis.

### Western blot analysis

Protein samples were applied into the sample wells in a sodium dodecylsulfate-polyacrylamide gel composed of 5% concentrated gel and 10% separation gel. The electrophoresis was first performed at 80 V for 45 min and then at 120 V until the electrophoresis run was complete. The gel slice containing the protein of interest was excised and the protein was transferred onto a polyvinylidene fluoride (PVDF) membrane at a constant current of 200 mA for 1.5 h. After being washed 3 times (each for 5 min) with a Tris-buffered saline-Tween 20 (TBST) buffer, the PVDF membrane was blocked with 5% skim milk powder in TBST buffer for 3 h at room temperature. The diluted primary antibody prepared with fresh blocking buffer was added and incubated overnight with rocking at 4 ^o^C. Then the membrane was washed 3 times (each for 10 min) with the TBST buffer, and the diluted secondary antibody was added, followed by incubation at room temperature for 50 min. After the PVDF membrane was washed 3–5 times (each for 10 min) with the TBST buffer, the membrane was put into a developer and developed by using Luminata™ Western HRP Substrates (Merck Millipore, Germany) and scanned with a Quantity One analysis software (Bio-Rad, USA).

### Quantitative determination of dopamine

Quantitative determination of dopamine was performed using a fluorometric method described by Schlumpf et al. [32]. For establishment of a dopamine standard curve, different concentrations of standard dopamine were prepared and were oxidized for 3 min by addition of iodine solution. After the reaction was stopped by addition of 200 µL Na_2_SO_3_ prepared with 5 M NaOH, 100 µL 0.1 M acetic acid was added, followed by heating at 100 ^o^C for 6 min and centrifugation after cooling. The collected supernatant was tested with a fluorescence spectrophotometer (Model F97, Lengguang Tech, China). For fluorometric quantification of the extracellular and intracellular dopamine, respectively, the culture medium and PC12 cells were separated by centrifugation. The collected cells were lysed by treatment with freeze/thaw cycles and then centrifuged. The supernatant was collected for the determination of intracellular dopamine.

### Statistical analysis

The results were reproduced in independent experiments. At least three independent experiments were performed. The means and standard deviations (SD) for each group were calculated. Data are presented as the mean ± SD. Statistical analyses of differences between the treatment groups and the control were performed using a paired *t*-test. The differences were considered to be significant at *P* < 0.05.

### Electronic supplementary material

Below is the link to the electronic supplementary material.

Additional file 1

Additional file 2

Additional file 3

Additional file 4

## Data Availability

The transcriptome sequencing raw data reported in this study were deposited in Sequence Read Archive (SRA) database with the accession link: https://dataview.ncbi.nlm.nih.gov/object/PRJNA843594?reviewer=apf90gf4gklnsv61s2ulplcvop. Accession Number: PRJNA843594(SAMN28771316、SAMN28771317) BioProject Accession Number: PRJNA843594 BioSample Accession Number: SAMN28771316、 SAMN28771317.

## Abbreviations

DETPs: differentially expressed transcript-encoded proteins
DMEM: Dulbecco’s modified Eagle’s medium
FBS: fetal bovine serum
LDH: lactate dehydrogenase
LETX-VI: Latroeggtoxin-VI
PVDF: polyvinylidene fluoride
Synj1: synaptojanin 1
