# Supplementary material for: Analysis of differentially expressed genes discovers Latroeggtoxin VI-induced changes and SYNJ1 as a main target in PC12 cells
Source: BMC Genomics. 2023 Sep 4;24:517. doi: 10.1186/s12864-023-09634-5 (PMC10478359; doi:10.1186/s12864-023-09634-5)
Supplement: Supplementary file 1 — Additional file 1 [file 12864_2023_9634_MOESM1_ESM.docx]

**Additional file 1.** Overall quality of the sequencing data.

| **Item** | **DZ 1** | **DZ 2** | **DZ 3** | **CL 1** | **CL 2** | **CL 3** |
| --- | --- | --- | --- | --- | --- | --- |
| Clean reads | 56926270 | 56332206 | 49191642 | 62340660 | 55761484 | 56253262 |
| Clean bases (kb) | 8270653 | 8119337 | 7135630 | 8978955 | 8082496 | 8145673 |
| Average read length (bp) | 145.29 | 144.13 | 145.06 | 144.03 | 144.95 | 144.8 |
| Q10 bases ratio (%)^a^ | 99.97 | 99.97 | 99.98 | 99.97 | 99.98 | 99.98 |
| Q20 bases ratio (%)^a^ | 99.22 | 99.09 | 99.27 | 99.12 | 99.32 | 99.31 |
| Q30 bases ratio (%)^a^ | 97.12 | 96.72 | 97.3 | 96.8 | 97.43 | 97.39 |
| N bases ration (%) ^b^ | 0 | 0 | 0 | 0 | 0 | 0 |
| GC bases ratio (%) | 53.45 | 53.34 | 53.24 | 53.64 | 53.21 | 53.69 |

^a^ Q10, Q20 and Q30 indicate that the correct rates of base recognition were 90%, 99% and 99.9%, respectively. ^b^ indicates the unknown bases. DZ, control; CL, LETX-VI treatment. n=3.
