# Supplementary material for: Analysis of differentially expressed genes discovers Latroeggtoxin VI-induced changes and SYNJ1 as a main target in PC12 cells
Source: BMC Genomics. 2023 Sep 4;24:517. doi: 10.1186/s12864-023-09634-5 (PMC10478359; doi:10.1186/s12864-023-09634-5)

**Additional file 4:**  Uncropped full-length blots

1. Uncropped full-length blots for those in Fig. 7A


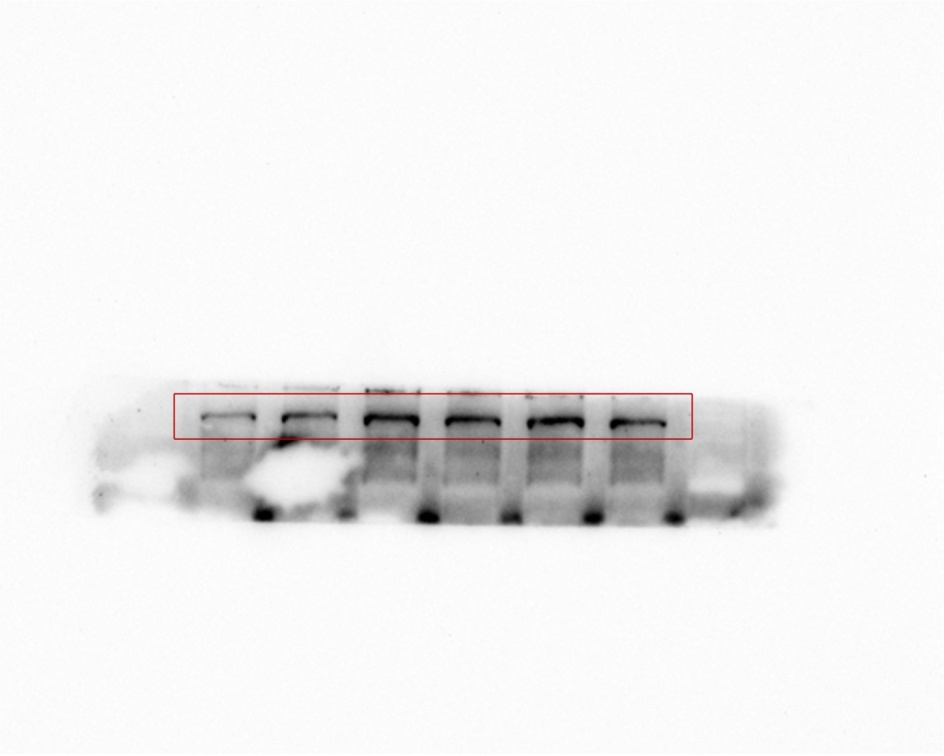


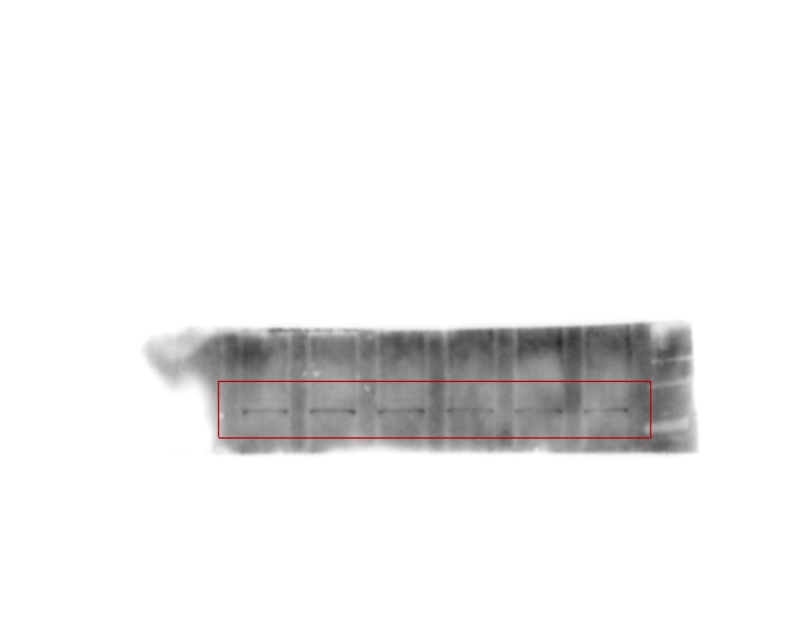


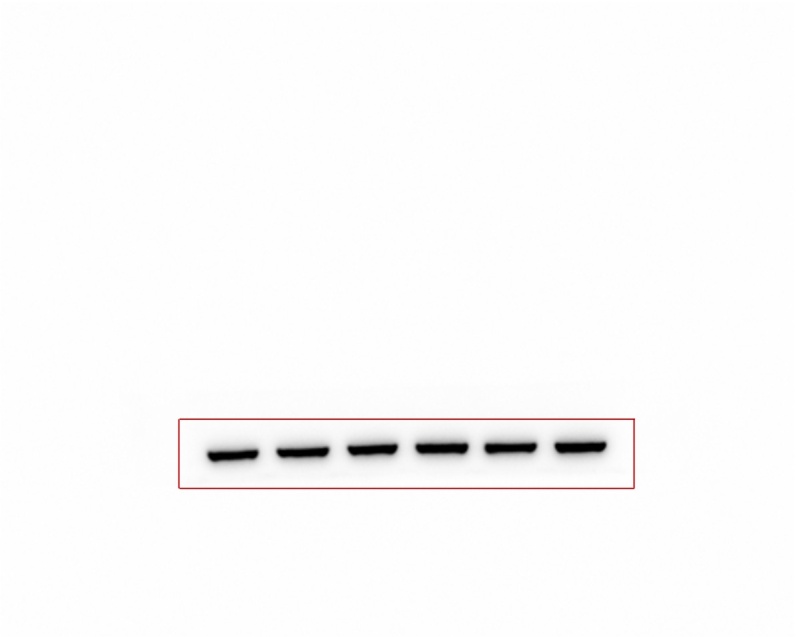


2. Uncropped full-length blots for those in Fig. 7D


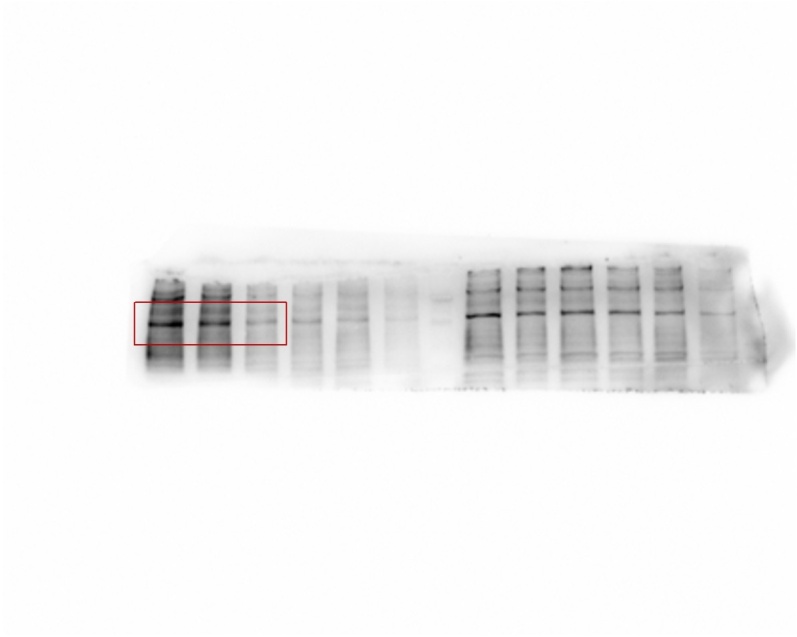


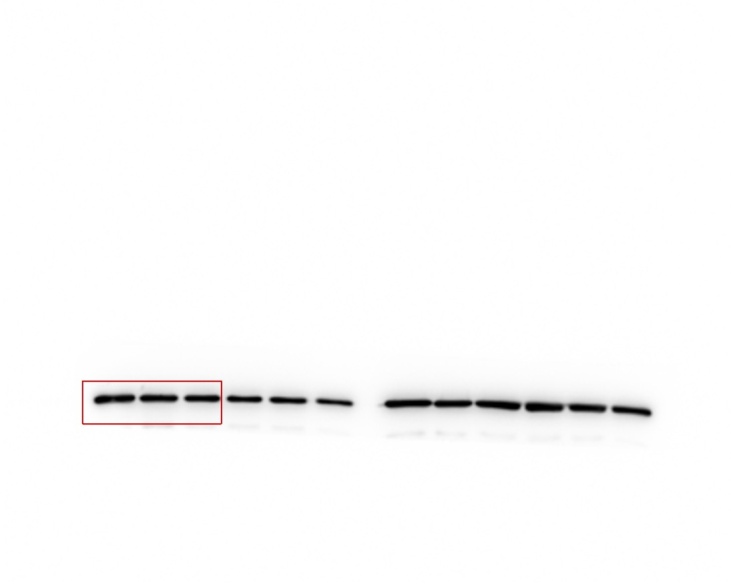


3. Uncropped full-length blots for those in Fig. 7F


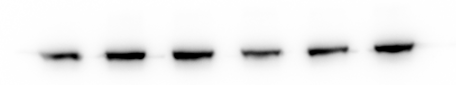


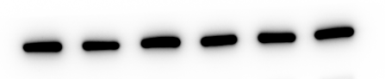

Supplement: Supplementary file 4 — Additional file 4 [file 12864_2023_9634_MOESM4_ESM.docx]
